# Classic infantile‐onset Pompe disease with histopathological neurologic findings linked to a novel *GAA* gene 4 bp deletion: A case study

**DOI:** 10.1002/mgg3.1957

**Published:** 2022-05-09

**Authors:** Magdalena Cerón‐Rodríguez, Daniela Castillo‐García, Carlos‐Patricio Acosta‐Rodríguez‐Bueno, Jesús Aguirre‐Hernández, Juan‐Rafael Murillo‐Eliosa, Pedro Valencia‐Mayoral, Argelia Escobar‐Sánchez, Juan‐Luis Salgado‐Loza

**Affiliations:** ^1^ Department of Lysosomal Diseases Hospital Infantil de México Federico Gómez Ciudad de México Mexico; ^2^ Department of Pediatrics Hospital Infantil de México Federico Gómez Ciudad de México Mexico; ^3^ Laboratory of Genomics, Genetics and Bioinformatics, Department of Genetics Hospital Infantil de México Federico Gómez Ciudad de México Mexico; ^4^ Department of Pathology Hospital Infantil de México Federico Gómez Ciudad de México Mexico; ^5^ Scientific Information Department SICODIC Mexico City Mexico

**Keywords:** acid alpha‐glucosidase, c.2066_2069delAGCC, *GAA* gene, infantile‐onset Pompe disease, neurologic, p.Glu689Glyfs*6

## Abstract

Pompe disease (PD) is an autosomal recessive disorder by a deficiency of acid α‐glucosidase (GAA) with intralysosomal glycogen accumulation in multiple tissues. We present the case of a 5‐month‐old male with hypertrophic cardiomyopathy, hypotony, feeding difficulties, and oxygen requirement since birth. At 3 months of age, he develops heart failure, respiratory impairment, and neurological deterioration. The echocardiogram revealed concentric hypertrophic cardiomyopathy with left‐diastolic dysfunction. We found increased creatine‐phosphokinase, lactate dehydrogenase, and urinary glucose tetrasaccharide levels, 50% of PAS‐positive vacuolated lymphocytes in the peripheral blood smear, and low GAA activity. Sequencing of coding exons and flanking intronic sequences revealed a novel homozygous 4 bp deletion in exon 15 of the *GAA* gene (c.2066_2069delAGCC/p.Glu689Glyfs*6). IOPD was diagnosed. At 5 months old, we started enzyme replacement therapy with an alpha‐alglucosidase of 20 mg/kg weekly and immunomodulation with intravenous immunoglobulin. He developed two cardiorespiratory arrests with subsequent neurologic deterioration, convulsive crisis, and respiratory failure and died at 9 months old. We found the usual PD hallmarks in the heart, striated muscle, and liver but also we found neuronal lesions characterized by cytoplasm vacuolization with PAS‐positive granules in the central nervous system and myenteric plexus. We describe a novel *GAA* gene pathogenic variant with a particular phenotype characterized by classic IOPD and neurologic histopathological findings. Enhancing the knowledge of lysosomal diseases is critical to improving the diagnosis and treatment of these patients.

## Graphical abstract

We report a patient with a novel GAA gene pathogenic variant (homozygous 4bp deletion in exon 15, c.2066_2069delAGCC/p.Glu689Glyfs*6) linked to a phenotype characterized by the presence of manifestations of Classic infantile‐onset Pompe disease with neurologic histopathological findings. Enhancing the knowledge of lysosomal diseases is critical to improving the diagnosis and treatment of these patients.

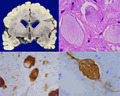

## 1 INTRODUCTION

Pompe disease (PD, OMIM # 232300) is an autosomal recessive disorder caused by a deficiency of alpha‐glucosidase in lysosomes (GAA, NP_000143.2, NM_000152.5, EC 3.2.1.20), coded by the *GAA* gene (GenBank reference sequence: NG_009822.1, HGNC: 4065, OMIM # 606800, Chromosomal location: 17q25.3) that results in intralysosomal glycogen (ChemIDplus 0009005792) accumulation in multiple tissue types, notably cardiac, skeletal, and smooth muscle. The severity of the disease depends on the amount of residual enzyme activity; in classic infantile‐onset of Pompe disease (IOPD), GAA activity is less than 1%, resulting in rapidly progressive hypertrophic cardiomyopathy (HCM), profound skeletal muscle weakness, and death usually within the first 12 months of life (Hirschhorn & Reuser, 2001; Van den Hout et al., 2003). Usually, the patients start with disease symptoms at 3 months of age and their death occurs at a median age of 6–8.7 months (Kishnani et al., 2010; Van den Hout et al., 2003). Many patients manifest cardiac problems, hypotonia and muscle weakness, respiratory distress, feeding difficulties, and failure to thrive at a median age of ~4 months, but neurologic involvement is much less frequent (Kishnani et al., 2006). Previous reports by Kishnani et al. (2006), Korlimala et al. (2019), Pena et al. (2015), and Teng et al. (2004) described neuronal and glial glycogen accumulation in PD patients with long‐term survival.

Lysosomes and their contents are involved in the pathogenesis of PD (Lim et al., 2014) and can be studied under the electron microscope as well as with several morphologic, histochemical, and conventional immunohistochemical techniques using antibodies directed to lysosomal proteins as lysosomal‐associated membrane protein 1 (LAMP1), lysosomal integral membrane protein 2 (LIMP2), and cathepsin D, and the glycogen stored in lysosomes can be highlighted with PAS stain (Koo & Pyel, 2021).

Enzyme replacement therapy (ERT) with recombinant human acid alpha‐glucosidase (rhGAA) has been commercially available since 2006 and led to improved clinical outcomes measures that include prolonged overall and ventilator‐free survival in IOPD patients (Banugaria et al., 2013), allowing the emergence of a new phenotype in survivors due to long‐term survival IOPD patients, mainly in those who have low titers of anti‐rhGAA IgG antibody titers (Prater et al., 2012). GAA protein is called cross‐reactive immunological material (CRIM) because anti‐GAA antibodies recognize it in Western blot analysis (Khallaf et al., 2012). ERT may lead to the production of anti‐drug antibodies, which negatively affect the safety and efficacy of this therapeutic option mainly in IOPD CRIM‐negative patients, who have no production of the enzyme at all, while CRIM‐positive patients have a nonfunctional form of GAA (Hahn & Schänzer, 2019), where high and sustained IgG antibodies (HSAT) has been reported, leading to a reduction in treatment efficacy (Desai et al., 2019). To avoid this outcome, an immunomodulatory regime based on Rituximab (RTX), Methotrexate (MTX), and intravenous immunoglobulin (IVIG) has successfully developed to achieve immune tolerance to ERT in infantile‐onset Pompe disease (Banugaria et al., 2013; Desai et al., 2019).

Almost 500 pathogenic variants, 191 benign variants, and 666 variants of unknown significance have been reported in the *GAA* gene, and genotype–phenotype correlations have been explored (Kopanos et al., 2018). We describe a novel homozygous 4 bp deletion in exon 15 of the *GAA* gene (c.2066_2069delAGCC/p.Glu689Glyfs*6) in a patient with a phenotype characterized by classic IOPD associated with histopathological neurologic findings and inadequate response to ERT.

## 2 CASE REPORT

**FIGURE 1:**
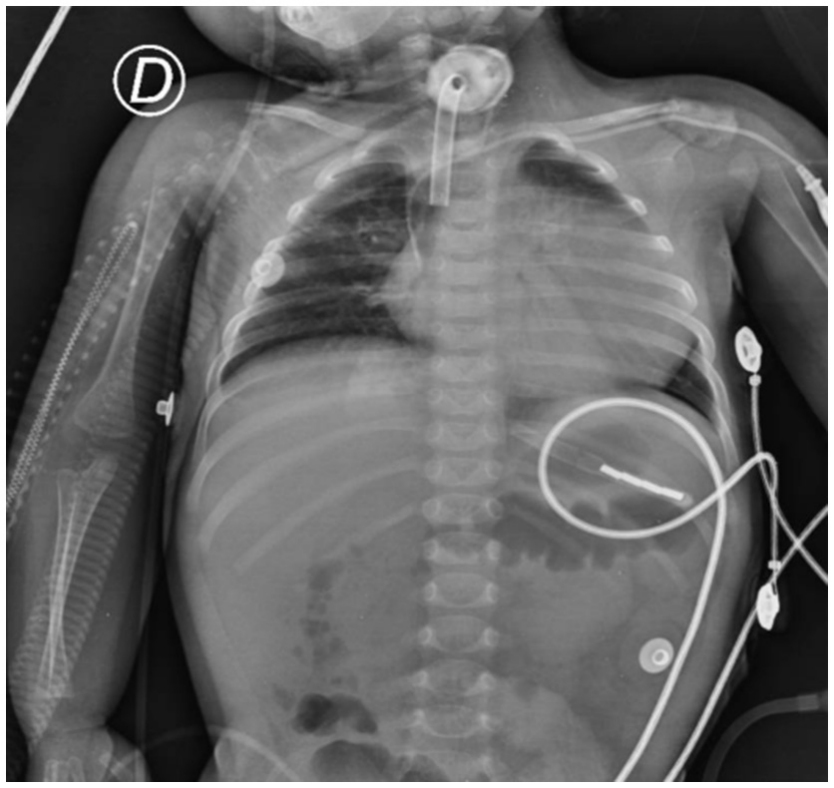
Chest X‐ray with cardiomegaly

The patient was a male born at full‐term by eutocic delivery from young, healthy, and presumably unrelated parents from Oaxaca, South México, with a healthy 2‐year‐old brother. After his birth, he required advanced cardiopulmonary resucitation with chest compressions at 3:1 rhythm and face mask lung inflation with 21% oxygen and pressure of 30 cm of water and remained hospitalized during his first 20 days of life, receiving supplemental oxygen. At discharge, the patient was hypotonic and had feeding difficulties. Three weeks later, at the age of 6 weeks, he was hospitalized in a Rural Hospital due to neonatal sepsis and showed generalized muscle weakness, hypotonia, feeding difficulties, macroglossia, cardiomegaly, and hepatomegaly. At 3 months of age, he developed breathing difficulties, heart failure, and neurological deterioration with hypoactive and hypotonic status requiring treatment with mechanical ventilation and diuretics. The echocardiogram revealed concentric hypertrophic cardiomyopathy with left‐diastolic dysfunction, left ventricular ejection fraction (LVEF) of 65%, and ventricular septal thickness of 10 mm. Thoracic X‐ray showed severe cardiomegaly (Figure 1). Laboratory tests showed increased creatine‐phosphokinase (453 UI/L; normal range: 24–170 UI/L) and lactate dehydrogenase (951 U/L; normal range: 91–180 U/L). The diagnosis of cardiac failure with hypotonia was made but PD was not suspected. At 5 months of age, due to poor response to treatment, he was referred to the Hospital Infantil de México, and we admitted him to the Department of Lysosomal Diseases with the suspicion of Pompe disease. We performed a dried blood spots test to assess GAA activity finding low levels (0.21 nmoL/ml blood/hr; normal range 1.29–5.7 nmoL/ml, Greenwood Genetic Center). Urine glucose tetrasaccharide (GLc4) level was 342.6 mmol/mg/creat (normal range: 0.14–1.29 mmol/mg/creat), and 50% of PAS‐positive vacuolated lymphocytes were found in the peripheral blood smear. We did not determine CRIM status because the analysis was not available. Amplification by a polymerase chain reaction and subsequent analysis with standard fluorescent sequencing protocol in both forward and reverse directions of all 19 coding exons of the *GAA* gene and immediate flanking intron sequences revealed a novel homozygous 4 bp deletion in exon 15 (c.2066_2069delAGCC), leading to frameshifting of the coding sequence and causing a predicted premature stop codon to appear six triplets downstream (p.Glu689Glyfs*6) (Greenwood Genetic Center).

We started ERT with a weekly regimen of alpha‐alglucosidase 20 mg/kg. We delayed immune tolerance induction (ITI) with RTX and MTX because he had *Pseudomonas aeruginosa* pneumonia. We only used a high‐dose, high‐frequency IVIG scheme to reduce the production of anti‐rhGAA IgG antibody titer. After 5 weeks of ERT and ITI with IVIG, the anti‐rhGAA IgG antibody titer was 3200. After 2 months of ERT at 7 months of age, an improvement in ventricular septal thickness and left ventricular mass were documented (Table 1), and we withdrew mechanical ventilation. After 12 weeks of ERT and ITI with IVIG, the anti‐rhGAA IgG antibody titer increased to 6400, but the level of GLc4 decreased to 49 mmol/mg/creat. Due to the increase of anti‐rhGAA IgG antibody titer, we decided to start intravenous RTX (375 mg/m^2^) and subcutaneous MTX (0.4 mg/kg) and continue IVIG (400 mg/kg), in addition to alpha‐alglucosidase 20 mg every week. After 4 weeks of treatment, mechanical ventilation restarted because he had a cardiorespiratory arrest with subsequent neurologic deterioration, seizures, and respiratory deterioration. One week later, he presented an irreversible cardiorespiratory arrest and died at 9 months of age.

**TABLE 1:** Echocardiographic evolution with ERT and ITI regimen

Age	IVSd (mm)	LV mass (g/m^2^)
5 months (Pre‐ERT)	18	321.5
6 months (1 month ERT & IVIG)	15	276
7 months (2 months ERT & IVIG)	11	262
9 months (4 months ERT & Full ITI)	11.7	240

Abbreviations: ERT, enzyme replacement therapy; ITI, immune tolerance induction; IVIG, intravenous immunoglobulin; IVSd, septal wall thickness at end‐diastole; LV, left ventricle.

**FIGURE 2:**
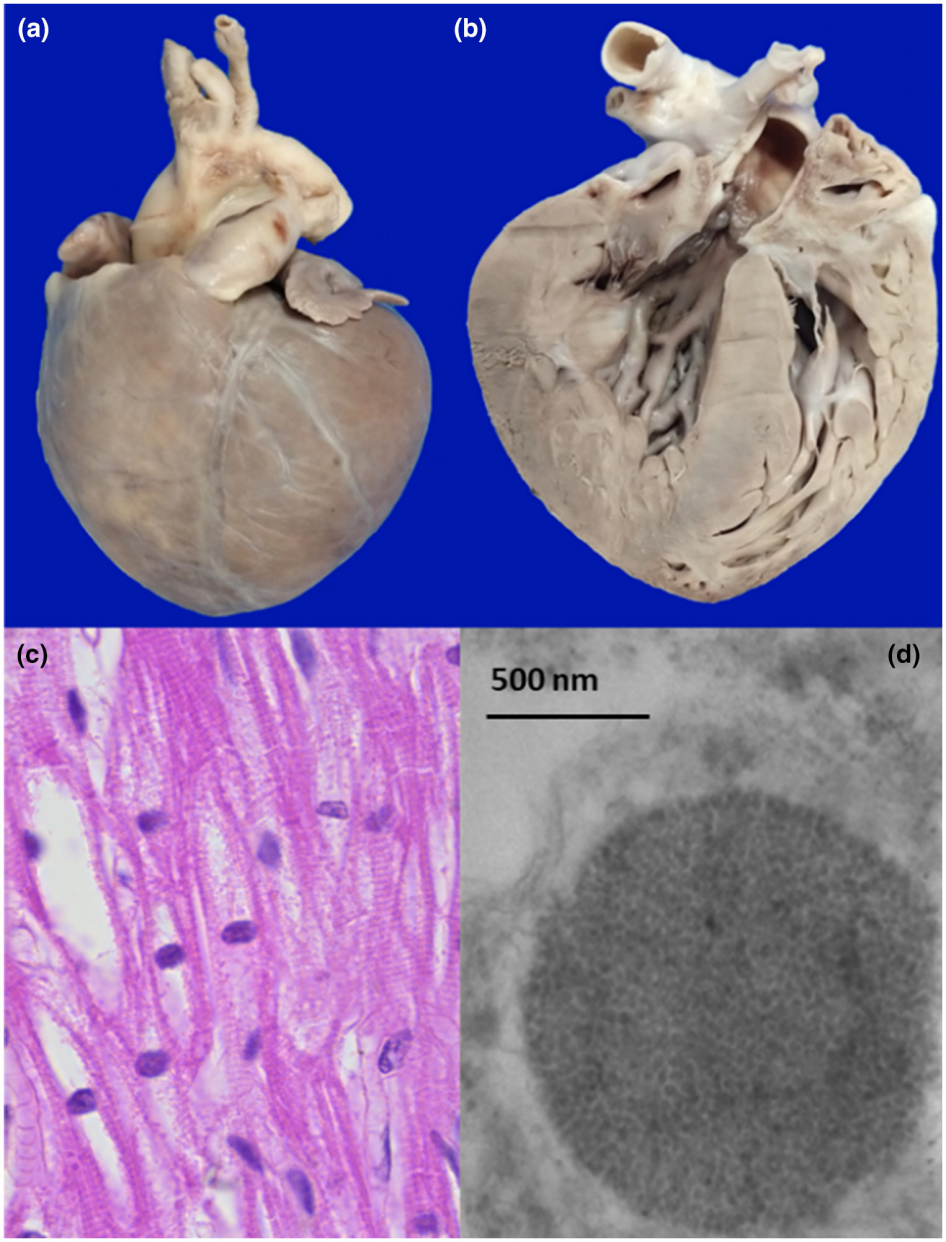
Heart: Panel a: The heart was enlarged and globoid‐shaped, panel b: On the cut section, the heart showed thickened ventricular walls, panel c: Cardiomyocytes with loss of striations, and microvacuolated cytoplasm (all H&E, original magnification × 100). Panel d: Ultra‐structurally, lysosomes‐containing mono‐particulate glycogen were observed (EM; original magnification × 10,000)

**FIGURE 3:**
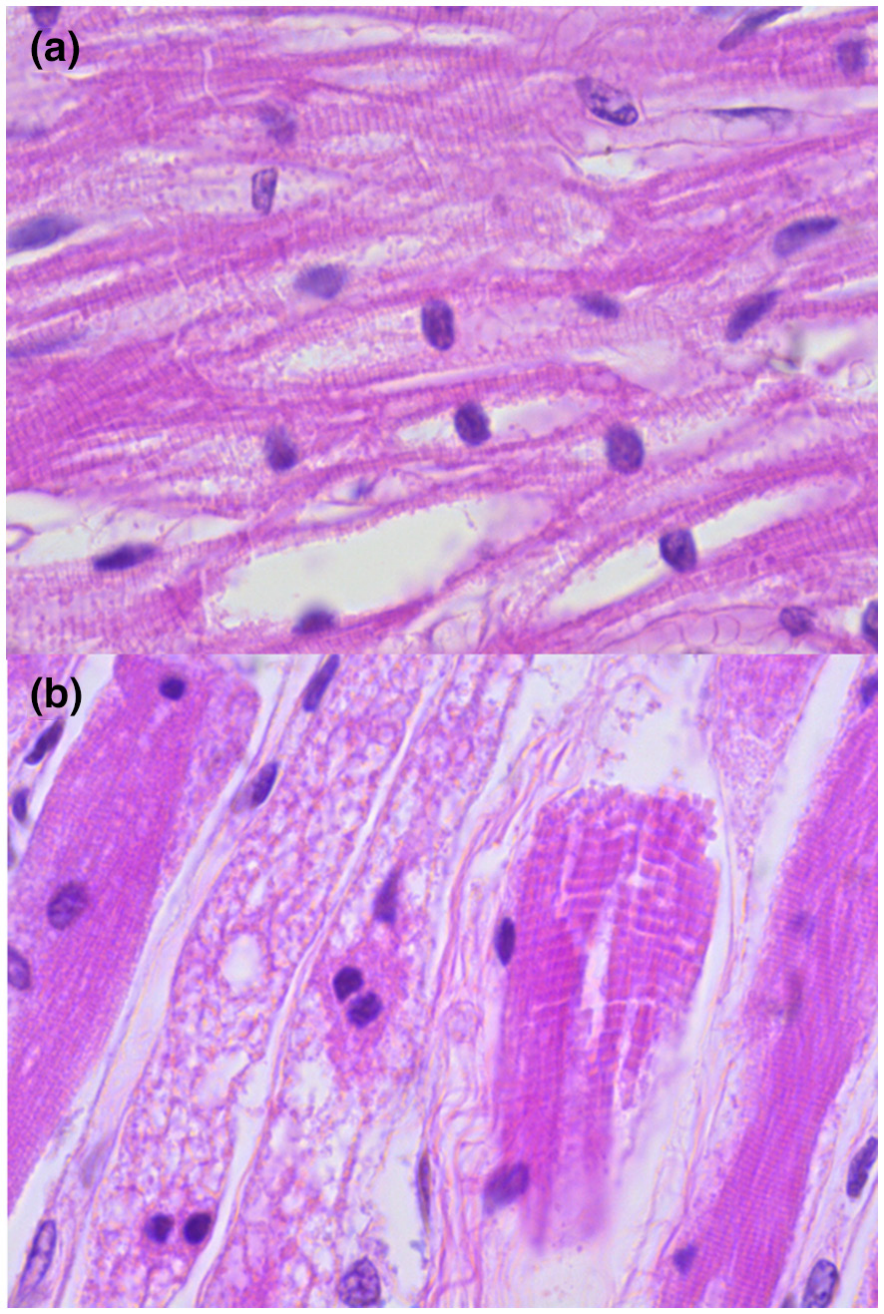
Muscle: Panel a: Extensive cytoplasm vacuolization of muscle cells and loss of striations of the tongue (H&E; original magnification × 40), panel b: Similar changes in the diaphragm and the major psoas muscle (H&E; original magnification × 100)

**FIGURE 4:**
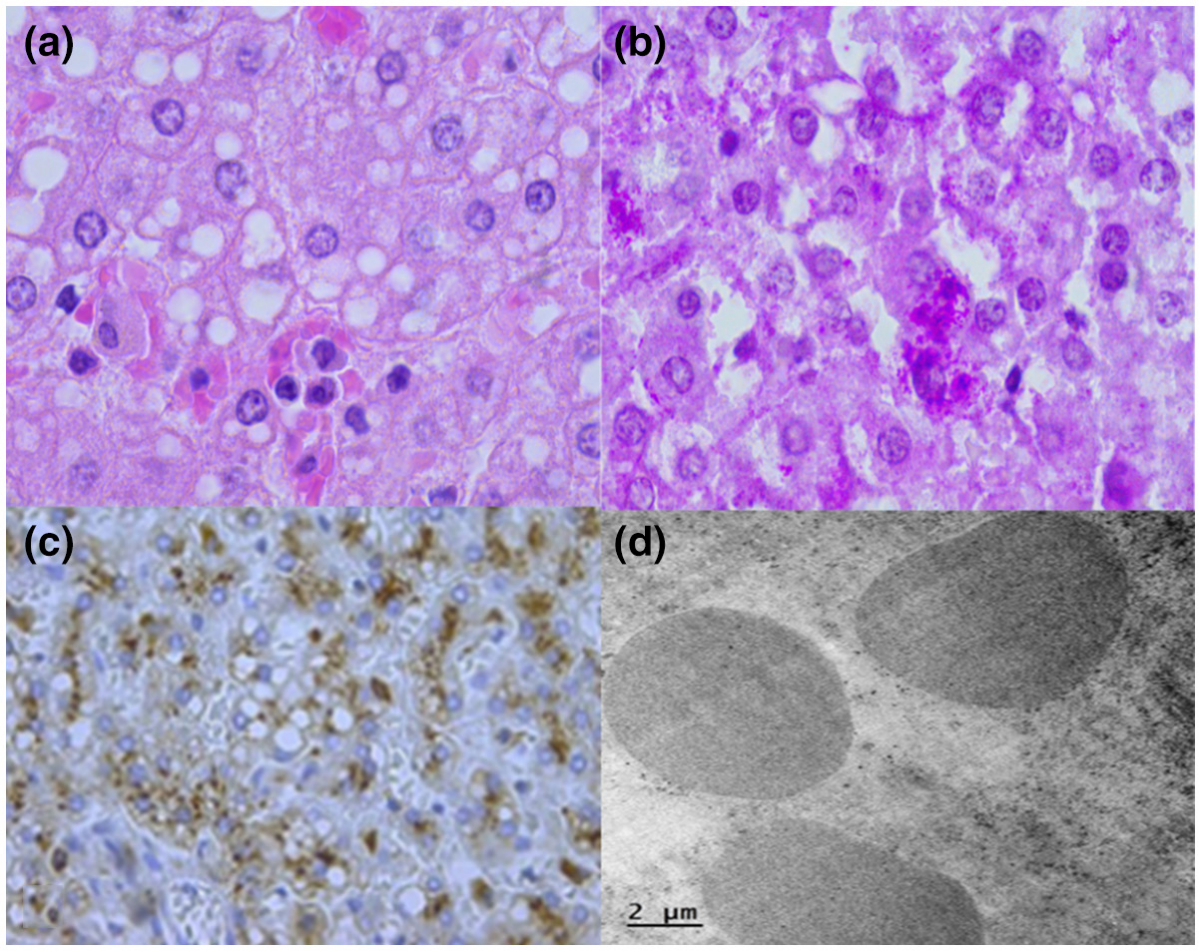
Liver: Panel a: Hepatocytes with microvesicular steatosis and granular cytoplasm (H &E, original magnification × 40), panel b: Cytoplasmic PAS‐positive granules (PAS stain, original magnification × 40), panel c: Cathepsin D was positive in the cytoplasm of hepatocytes (original magnification × 20), panel d: Lysosomes‐containing granular glycogen (electronic microscopy. Original magnification × 5000)

Postmortem examination showed findings mainly in the heart, striated muscle, liver, and nervous system. Macroscopically, we saw an enlargement of the heart (48 g vs. an expected weight of 37 g) with a globoid shape (Figure 2a) and thickening of ventricular walls (Figure 2b). Microscopically, we observed extensive damage to the cardiomuscular cells characterized by empty‐looking cytoplasm, loss of muscular striations, and multiple microvacuolization (Figure 2c). At electron microscopy (EM), several lysosomes‐containing mono‐particulate glycogen were observed (Figure 2d). We found similar changes with extensive cytoplasm vacuolization in the striated muscle of the tongue, psoas, and diaphragm (Figure 3). The liver was enlarged (300 g compared with an expected weight of 254 g), and histologically, it exhibited diffuse microvesicular changes (Figure 4a) and PAS‐positive granules (Figure 4b) in hepatocytes cytoplasm. We demonstrate that vacuoles were lysosomes using immunohistochemistry (Formalin/PFA‐fixed paraffin‐embedded section) on liver samples with antibodies directed to lysosome‐associated proteins: LAMP1, LIMP2, and CD (Abcam Laboratory, USA; catalog number 62562 [unconjugated rabbit polyclonal antibody to LAMP1], 176317 [unconjugated rabbit monoclonal antibody to LIMP2], and 75852 [unconjugated rabbit monoclonal antibody to CD]) (Figure 4c). The EM study of the liver showed that some vesicles were lipid droplets, whereas others corresponded to lysosomes with mono‐particulate glycogen (Figure 4d).

**FIGURE 5:**
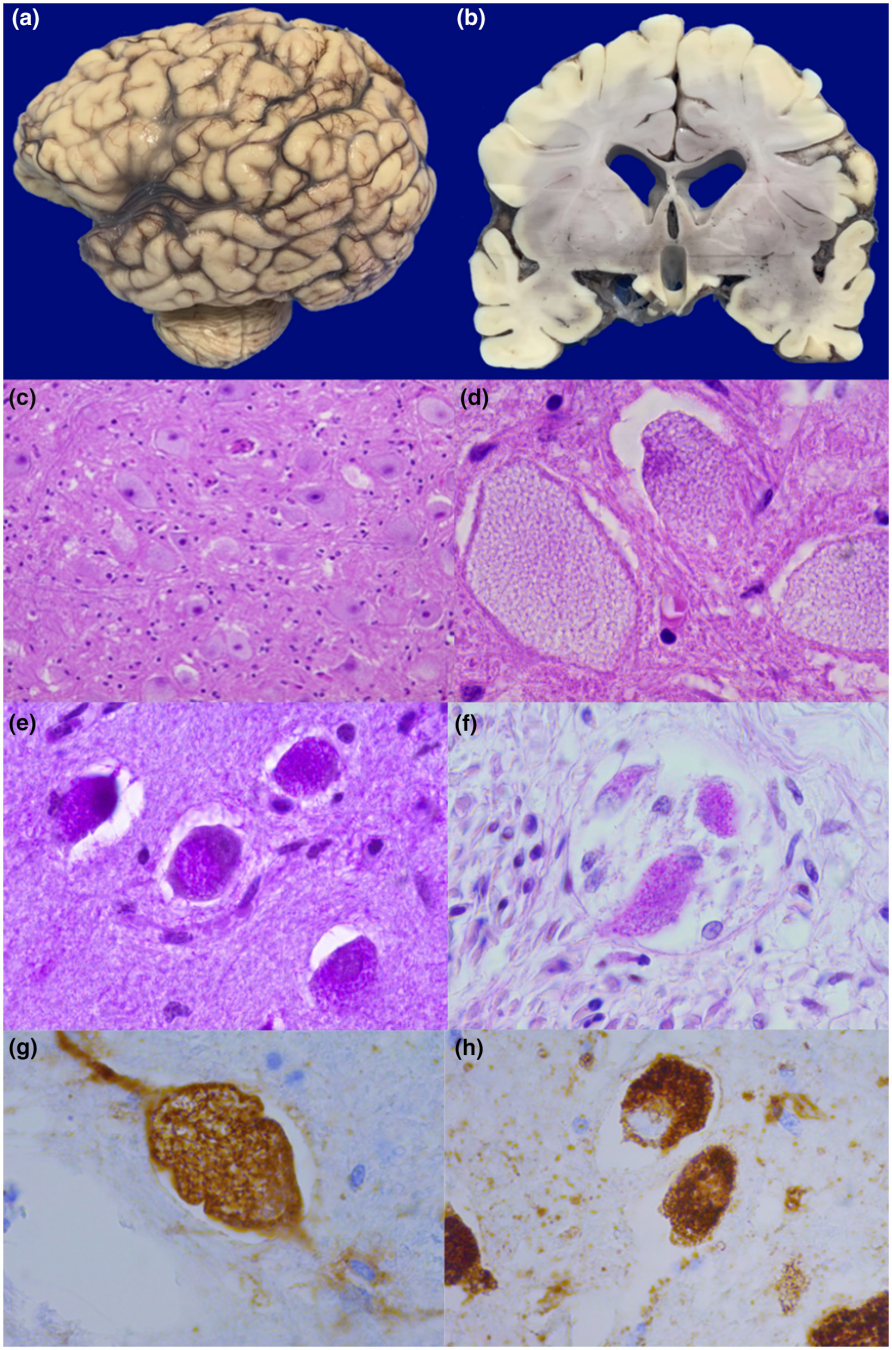
Brain: Panels a and b: Encephalic atrophy characterized by deep brain grooves and dilatation of ventricles, panels c and d: Enlarged neurons with prominent vacuolization of the cytoplasm, panels e and f: Vacuoles containing PAS‐positive material. Panel g and h: Cathepsin D (g) and lysosomal integral membrane protein (h) reveal that vacuoles correspond to lysosomes

We observed severe cerebral atrophy (weight 400 g vs. an expected weight of 714 g) (Figure 5a,b). Macroscopically, the cerebellum was also atrophic. Microscopically, extensive neuronal damage was present in two forms: the first damage was associated with hypoxic cortical neuronal changes that affect from frontal lobes to Purkinje cells in the cerebellum. The second type of neuronal lesions was cytoplasm vacuolization (Figure 5c,d) with vacuoles containing PAS‐positive granules. Neurons in the basal ganglia, mesencephalon, pons, and spinal medulla exhibited cytoplasm vacuolar transformation, and the same findings in ganglionic neurons of the myenteric plexus of both, small intestine and colon were observed. We also performed immunohistochemistry (Formalin/PFA‐fixed paraffin‐embedded section) with LAMP 1, LIMP 2, and CD antibodies (Abcam Laboratory, USA; catalog number 62562 [unconjugated rabbit polyclonal antibody to LAMP1], 176317 [unconjugated rabbit monoclonal antibody to LIMP2], and 75852 [unconjugated rabbit monoclonal antibody to CD]). We found that vacuoles in neurons and glial cells were lysosomes (Figure 5g,h). The remaining tissues and organs presented several changes due to hypoxia‐ischemia and shock such as cellular damage (karyorrhexis, pyknosis, and apoptosis), a diverse degree of tiss necrosis, interstitial edema, vascular congestion, and focal hemorrhages.

## 3 DISCUSSION

The manifestations and clinical course of our patient correspond with a classical form of IOPD not only in the presence of multi organic damage but also at the beginning of his alteration, the time of diagnosis, appearance of symptoms, and survival age reported by Kishnani et al., who found the median age of diagnosis at 4.7 months, the median age of death at 8.7 months, and the appearance of symptoms at 3 months of age (Kishnani et al., 2006), linked to a novel homozygous 4 bp deletion (c.2066_2069delAGCC/p.Glu689Glyfs*6) found in exon 15 of *GAA* gene. Sadly, a delay in his diagnosis resulted from the limited resources and the lack of doctors’ experience with this kind of patient because the initial treatment was in a rural hospital, and we diagnosed PD until we received him at our hospital. Also, the disease severity, the presence of lysosomal glycogen deposits in neurons, and poor treatment response could be associated with the delay at the beginning of the treatment because establishing the relationship between c.2066_2069delAGCC/p.Glu689Glyfs*6 variant and the clinical presentation of the classical form of IOPD plus neurologic involvement are not feasible. Our case highlights the need for more knowledge of orphan diseases, where an early start of treatment implies a change in the evolution and prognosis of the disease.

The resulting frameshift in the coding sequence and the creation of a premature stop codon, six codons ahead, would remove the end of the catalytic GH31 domain, plus the proximal and distal beta domains at the carboxy end of the protein (Roig‐Zamboni et al., 2017), representing almost a fifth of the full‐length polypeptide. It is expected that this would prevent the protein from being normally processed and folded and would likely be degraded. In our patient, the pathogenic homozygous genotype is consistent with the features of early onset Pompe disease: namely, the disease onset before the age of 12 months, cardiomyopathy, and low enzymatic activity.

Homozygous frameshift and nonsense variants downstream of the one being reported here and leading to premature stop codons closer to the carboxy terminus of the protein were reported in individuals with the infantile form of the disease: Leu729TrpfsTer35, Tyr773fsTer3, Arg854Ter, Arg870Ter, Glu888Ter, and Gln914fsTer30 (Alansari et al., 2013; Becker et al., 1998; Hermans et al., 1997, 1998; Herzog et al., 2012; McCready et al., 2007; Oba‐Shinjo et al., 2009; Pereira et al., 2008; Pittis et al., 2008; Reuser et al., 2019). The association of these homozygous variants with the severe presentation of Pompe disease underscores the relevance of the carboxy end of the protein (proximal and distal beta domains) for the proper processing, folding, and functioning of the enzyme.

Even though the particular variant that we describe has been not reported in Pompe patients previously (Peruzzo et al., 2019; Reuser et al., 2019), nor disease databases (Fokkema et al., 2011; Landrum et al., 2016; Stenson et al., 2017), or exome or genome databases (Karczewski et al., 2020; Lek et al., 2016). The *GAA* 689 residue is well known for containing the nonpathogenic Glu689Lys variant (Huie et al., 1996). This variant is linked, in Asian populations, to the pseudo‐deficiency variant Gly576Ser, which shows reduced catalytic activity although it does not lead to disease (Kroos et al., 2008).

We performed the anatomopathological study of the pathological (gross, histological, immunohistochemical, and ultrastructural) findings in the heart, striated muscle, and liver in our patient, where the changes usually described in Pompe disease were present (Burt et al., 2017; Lynch et al., 2005). Also, we found a neurologic involvement manifested by cytoplasmic LAMP1, LIMP2, and cathepsin D vacuolization in neurons of the basal ganglia, mesencephalon, pons, spinal medulla, and ganglionic neurons of the myenteric plexus due to lysosomal glycogen storage. In previous reports, neurologic involvement was described in 9.5% of the patients with IOPD, mainly as localized or generalized brain atrophy (Kishnani et al., 2006). Kourlimarla et al. reviewed the evidence of the neurologic damage produced by PD and proposed a new phenotype existence as the result of longer survival of patients with PD due to the use of ERT with the involvement of the central nervous system. However, its clinical impact is still not well understood (Korlimarla et al., 2019). Various neurological alterations have been clinically described in patients with IOPD, like neurological motor symptoms and cognitive deficiencies (Byrne et al., 2019; Chien et al., 2006), but correlating them with the histopathological findings is difficult because the muscular approach to the disease excludes the neurological examination. Our patient had neurologic damage due to hypoxia and lysosomal vacuoles and cannot possibly establish a relation between them or make a clinical correlation to differentiate if the neurologic deficits correspond to hypoxia or lysosomal glycogen storage. Pena et al. described three cases of IPD with extensive glycogen accumulation in neurons and glial cells of the white matter, brainstem, cerebellum, with relative sparing of cerebellar Purkinje cells, ganglion cells of the GI tract, adrenal glands, pancreas, and anterior horn of the spinal cord linked to homozygosity c.2560C>T, c.766_785del_ins_C (p.Tyr256fsX6), and c.2432delT (p.Leu811fsX37) variations (Pena et al., 2015). Teng et al. report one IOPD patient with marked neuronal ballooning changes with PAS‐positive stain only in the neurons of the spinal cord and medulla linked to Gly615Arg variation of the exon 13 (Teng et al., 2004).

## 4 CONCLUSION

We found in our patient a novel *GAA* gene pathogenic variant (homozygous 4 bp deletion in exon 15, c.2066_2069delAGCC/p.Glu689Glyfs*6), linked to a phenotype characterized by: (a) the presence of manifestations of classic IOPD with neurologic histopathological findings perhaps, seizures could be a symptom of neurological damage; (b) the poor response to ERT with development of anti‐rhGAA IgG antibody; and (c) a catastrophic clinical evolution. These characteristics could represent another phenotype of IOPD because of the involved organs and the response to the treatment. The variants determination in the *GAA* gene provides valuable information to identify phenotypes associated with specific variants and genotypes and provide early genetic counseling. Our case highlights the need for more knowledge of orphan diseases, where an early start of treatment implies a change in the evolution and prognosis of the disease.

## CONFLICT OF INTEREST

Magdalena Cerón‐Rodríguez, Pedro Valencia‐Mayoral, and Argelia Escobar Sánchez have received a fee for speaking from different pharmaceutical companies. Juan‐Luis Salgado‐Loza has received payment for writing assistance from pharmaceutical companies. Daniela Castillo‐García, Carlos Patricio Acosta‐Rodríguez‐Bueno, Jesús Aguirre‐Hernández, Juan Rafael Murillo Eliosa do not have any conflict of interest to declare. The authors confirm independence from pharmaceutical companies. The information of the article has not been influenced by pharmaceutical companies.

## AUTHOR CONTRIBUTIONS

Magdalena Cerón‐Rodríguez contributed to the conception, design, acquisition, analysis, and interpretation of data and review. Daniela Castillo‐García contributed to the conception, acquisition, analysis of data, and review. Carlos‐Patricio Acosta‐Rodríguez‐Bueno and Pedro Valencia‐Mayoral contributed to acquisition, analysis, interpretation, and review. Jesús Aguirre‐Hernández, Juan‐Rafael Murillo‐Eliosa, and Argelia Escobar‐Sánchez contributed to the analysis and interpretation of data and review. Juan‐Luis Salgado‐Loza contributed in conception, design, analysis of data, interpretation of data and drafting.

## ETHICS

This paper was approved by the ethics committee.

## Data Availability

The data that support the findings of this study are available from the Hospital Infantil de México Federico Gomez. Restrictions apply to the availability because are not public.
